# Atypical ovarian carcinoid tumor with widespread skeletal metastases: a case report of multiple endocrine neoplasia type 1 in a young woman

**DOI:** 10.1186/s12885-019-6332-7

**Published:** 2019-11-14

**Authors:** Lei Lou, Lixia Zhou, Wenyan Wang, Huina Li, Yuehong Li

**Affiliations:** 1 Department of Pathology, The Second Hospital of Hebei Medical University, Shijiazhuang City, Hebei Province 050000 People’s Republic of China; 2 Department of Radiology, The Second Hospital of Hebei Medical University, Shijiazhuang City, Hebei Province 050000 People’s Republic of China; 3 Department of Radiotherapy, The Second Hospital of Hebei Medical University, Shijiazhuang City, Hebei Province 050000 People’s Republic of China; 4 Department of Pathology, Hebei Maternity and child Healthcare Hospital, Shijiazhuang City, Hebei Province 050000 People’s Republic of China

**Keywords:** Multiple endocrine neoplasia type 1 syndrome, MEN1, Carcinoid tumor, Skeletal metastasis, Neuroendocrine tumors, Primary hyperparathyroidism

## Abstract

**Background:**

Multiple endocrine neoplasia type 1 (MEN1) is a rare autosomal dominant inherited condition affecting multiple endocrine organs, resulting in significant morbidity and decreased life expectancy. Early tumor identification allows for timely patient management, reduces morbidity, and improves disease outcomes. Patients with MEN1 typically present with primary hyperparathyroidism caused by multiple parathyroid tumors, however, thymic and bronchial carcinoid tumors are also less common manifestations. MEN1-related neuroendocrine tumors often show hematogenous metastasis, with the liver being the most common metastatic site. Skeletal metastases from neuroendocrine tumors are extremely rare.

As few as 50 case reports were identified in a recently published literature review on skeletal metastases from carcinoid tumors. To our knowledge, studies related to MEN1 have not been previously conducted.

**Case presentation:**

We present a case of MEN1-related atypical ovarian carcinoid presenting as the first disease manifestation in a 30-year old woman. After two years, another atypical carcinoid was incidentally diagnosed in the contralateral ovary during a caesarean section. Syndromic MEN1 was not diagnosed clinically despite her young age and bilateral involvement. The patient remained disease-free for two years without further adjuvant treatment prior to clinic presentation with complaints of chest discomfort and body pain. Radiologic and pathologic investigations identified multifocal simultaneous neuroendocrine tumors involving the parathyroid, thymus, pancreas, and adrenal glands, in addition to multiple other metastatic sites. The findings ultimately resulted in the patient being diagnosed with MEN1.

**Conclusions:**

This extremely rare case emphasizes that ovarian carcinoids, especially when bilateral, could be the initial manifestation of MEN1. The significance of this differential diagnosis was highlighted by the subsequent detection of widespread skeletal metastasis resulting from the carcinoid tumors. A low threshold of suspicion, systemic diagnostic work-up, and regular follow-up are of utmost importance to timely diagnosis of MEN1.

## Background

Multiple endocrine neoplasia type 1 (MEN1) is a complex, multisystem disease manifesting with a diverse range of primary and secondary metabolic and neoplastic disorders. Its prevalence is approximately 1–10/100000 with a high penetrance [1, 2]. MEN1 affects all age groups and clinical or biochemical manifestations develop in more than 94% of patients by the fifth decade [3–5]. A recent review of the Italian MEN1 registry revealed that the average age of MEN1 diagnosis was 55.1 years [4]. A comprehensive study showed that the overall predominance of female patients with MEN1 disease was 58% [6]. The clinical presentation of MEN1 usually manifests as occurrence of tumors in the parathyroid gland, anterior pituitary gland, and pancreatic islet cells, with less common occurrences that include adenomas of the adrenal glands and neuroendocrine tumors [7, 8]. Extensive evidence indicates that early diagnosis and intensive follow-up of MEN1 patients may decrease morbidity and improve life expectancy [9, 10]. Conversely, a delay in MEN1 diagnosis has been found to cause advanced metastatic neuroendocrine tumor manifestations [11]. Thus, a timely and accurate diagnosis is key to the management of MEN1. Both the relatively low incidence and the heterogeneous group of tumors that present with varying clinical manifestations have contributed to the paucity of large epidemiological studies characterizing the clinical presentations and disease courses for patients with MEN1. Herein, we present a case of MEN1-related atypical ovarian carcinoid presenting as the first disease manifestation in a 30 year-old woman. The study was approved by the Ethics Committee of The Second Hospital of Hebei Medical University, Shijiazhuang, China and the patient provided written informed consent for publication of this report.

## Case presentation

**Fig. 1:**
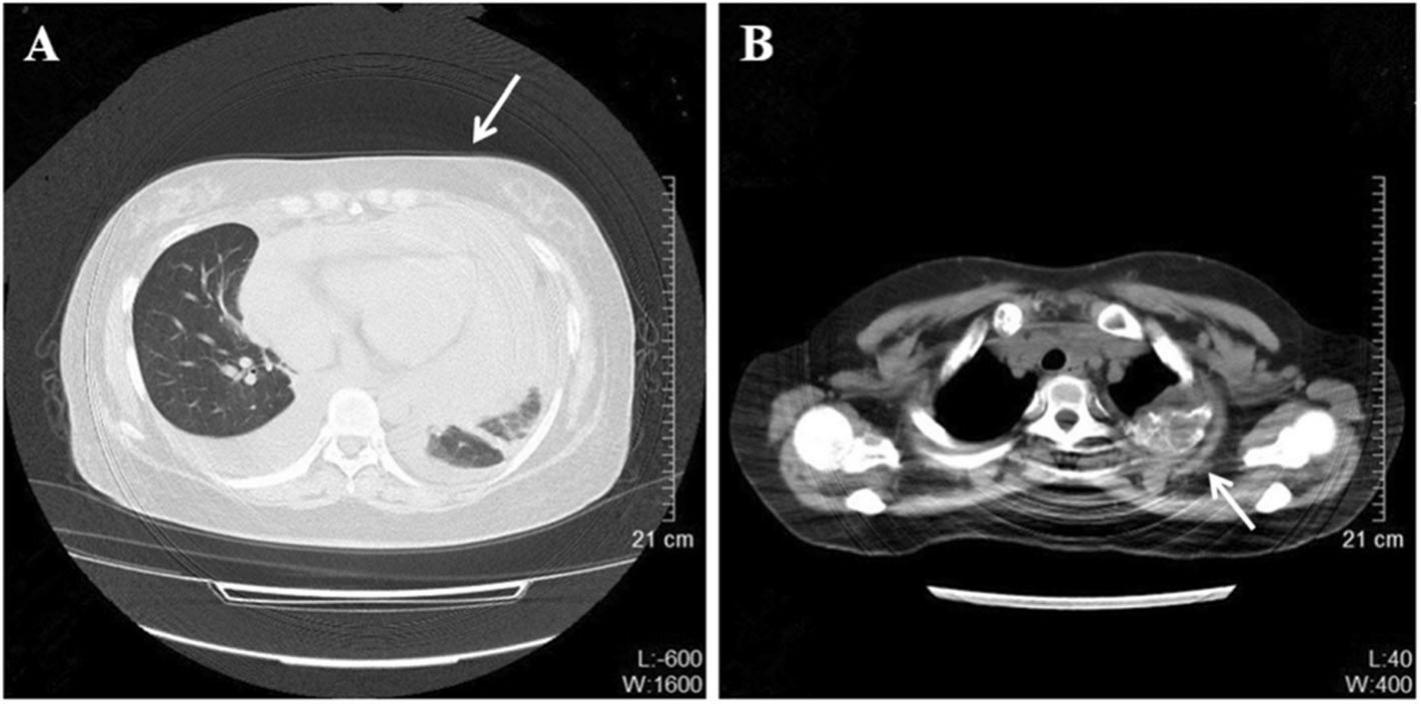
Chest computed tomography obtained at time of initial presentation showing massive pericardia and pleural fluid (white arrow) (**a**) in addition to pleural effusion and an osteolytic lesion on the second left rib (white arrow) (**b**)

**Fig. 2:**
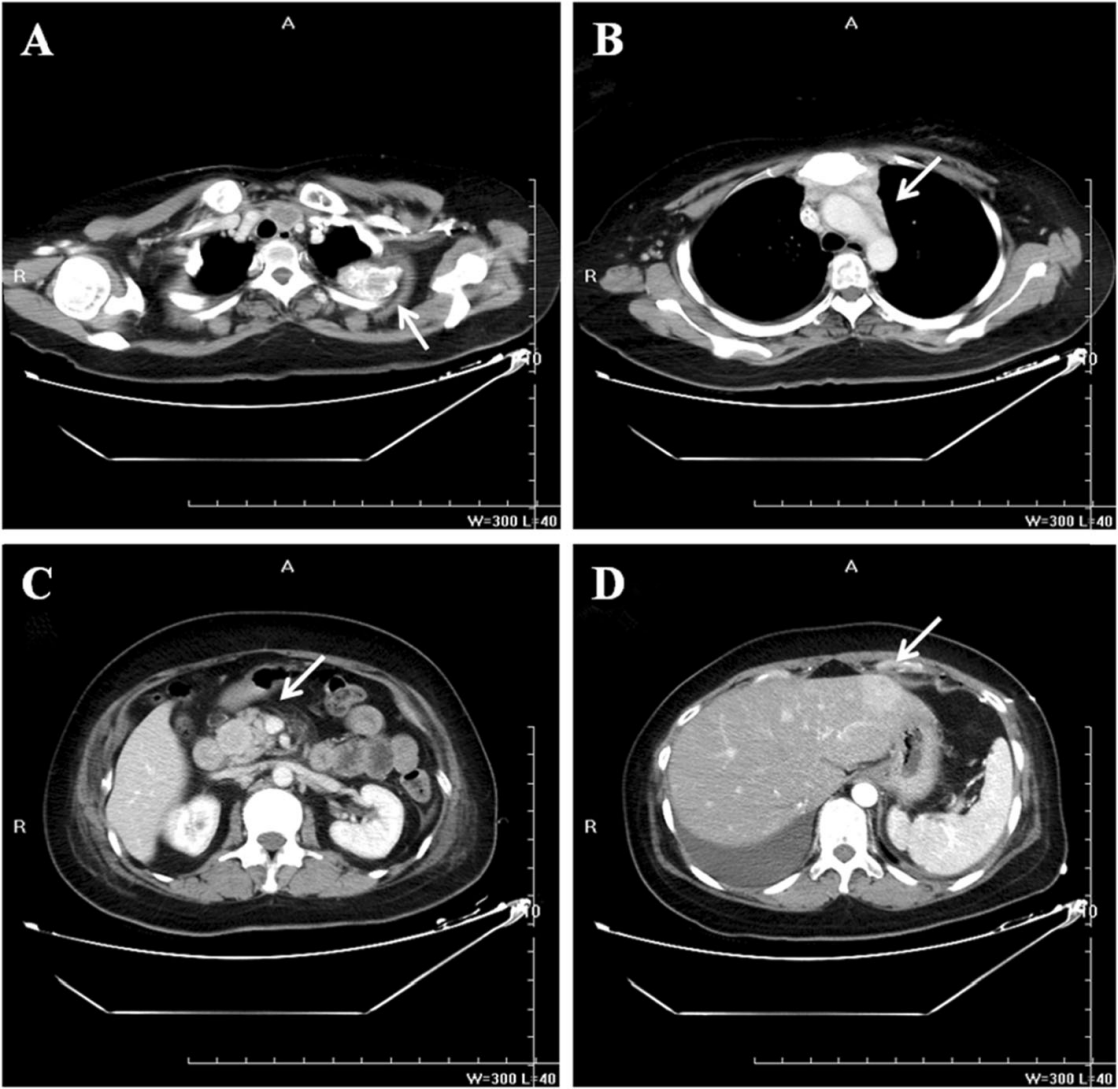
Contrast enhanced computed tomography scan of the upper abdomen show a heterogeneous mass (white arrow) and a high degree of enhancement in the ribs (**a**), anterior mediastinum (**b**), pancreas (**c**), and liver (**d**)

**Fig. 3:**
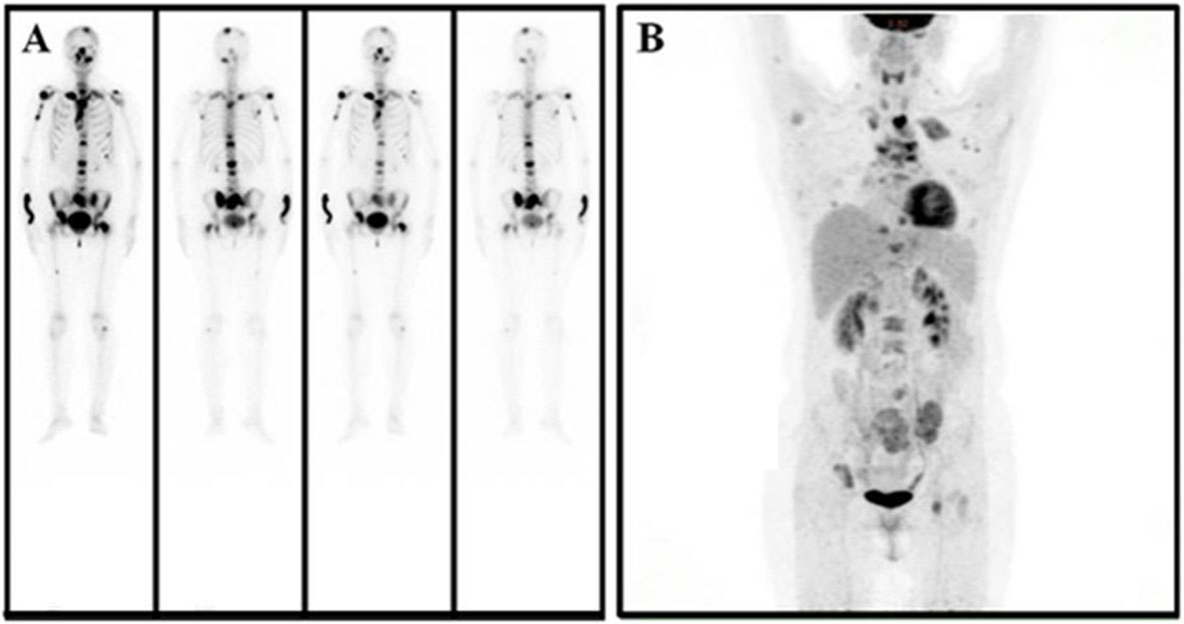
Bone scintigraphy and Positron emission tomography (PET) scans showed multiple metastatic lesions. (**a**) Bone scintigraphy showed increased uptake into multiple bones including the skull, cervical, thoracic and lumbar vertebrae and ribs, humerus, as well as the pelvis. (**b**) PET scans showing hypermetabolic parathyroid gland, thymus, lung, pancreas, adrenal gland, and multiple metastatic foci in the bones, liver and several lymph node regions

A 30-year-old woman underwent a left oophorectomy, after which she was diagnosed with atypical carcinoid of the ovary. Two years following this diagnosis, she underwent a caesarean section, and an atypical carcinoid in the contralateral ovary was incidentally found. After the atypical carcinoid was pathologically diagnosed, she remained disease-free for another two years. Subsequently, the patient was admitted to our hospital with complaints of chest discomfort resulting from pericardial fluid accumulation. The results of chest computed tomography (CT) showed massive pericardial fluid accumulation, pleural effusion, and an osteolytic lesion in the second rib on the left (Fig. 1). Contrast-enhanced CT images of the upper abdomen showed multivisceral spread of the neuroendocrine tumors and metastases to the bones and liver (Fig. 2). A whole-body bone scan showed increased uptake in multiple bones, including the skull, ribs, humerus, pelvis, as well as the cervical, thoracic, and lumbar vertebrae (Fig. 3a). Positron emission tomography-computed tomography (PET-CT) demonstrated hypermetabolic areas in the parathyroid gland, thymus, lung, pancreas, and adrenal glands with multiple metastatic foci in the bones, liver, and several lymph nodes (Fig. 3b).

**Fig. 4:**
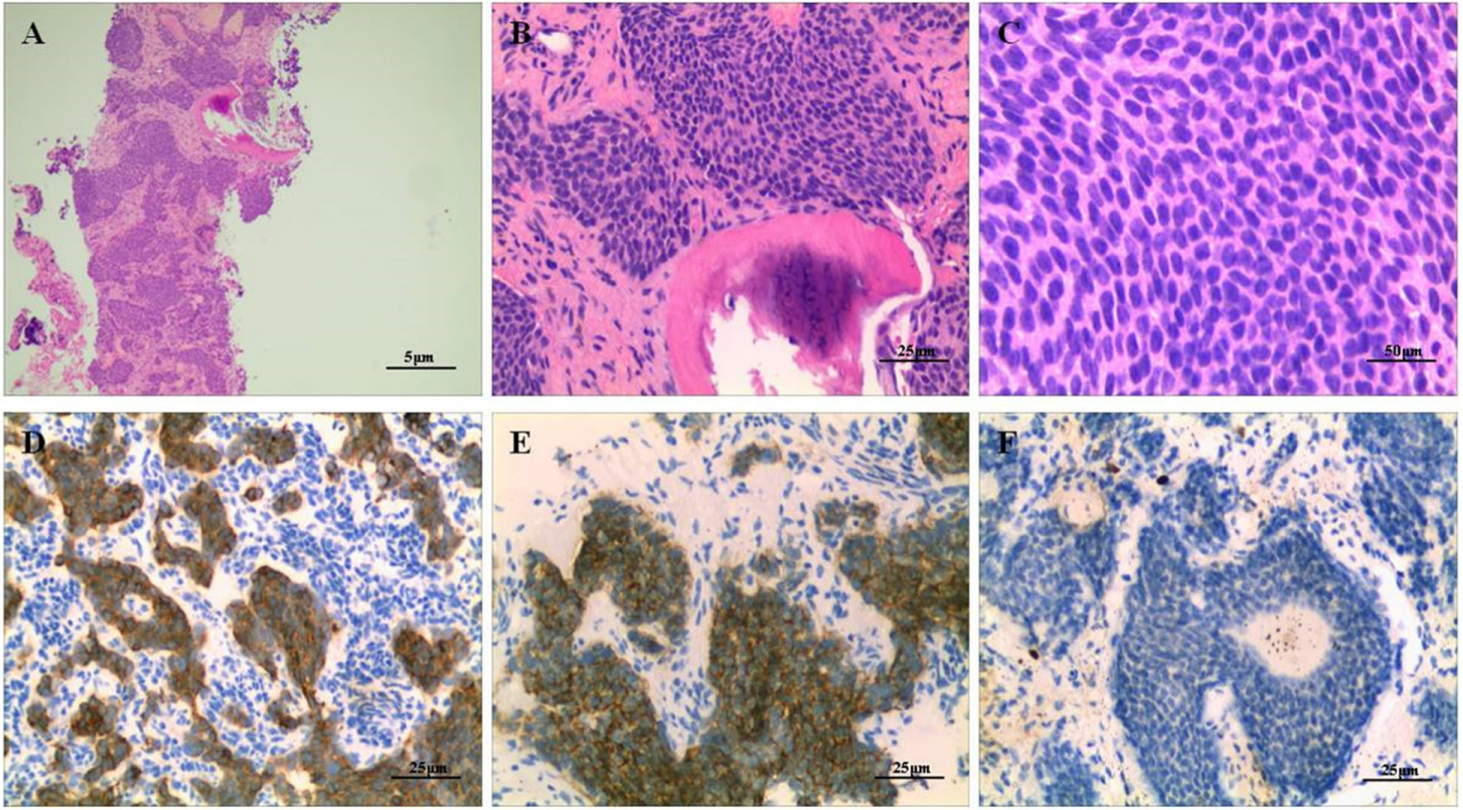
Histopathological examination of the needle biopsy (H&E staining) of the 2nd rib shows the tumor consisting of cellular nests of small round cells, no mitoses, original magnification, × 40 (**a**), × 200 (**b**), × 400 (**c**). These cells were positive for synaptophysin, original magnification, × 200 (**d**), CD56, × 200 (**e**) and Ki-67 was less 1% upon immunostaining, × 200 (**f**)

**Fig. 5:**
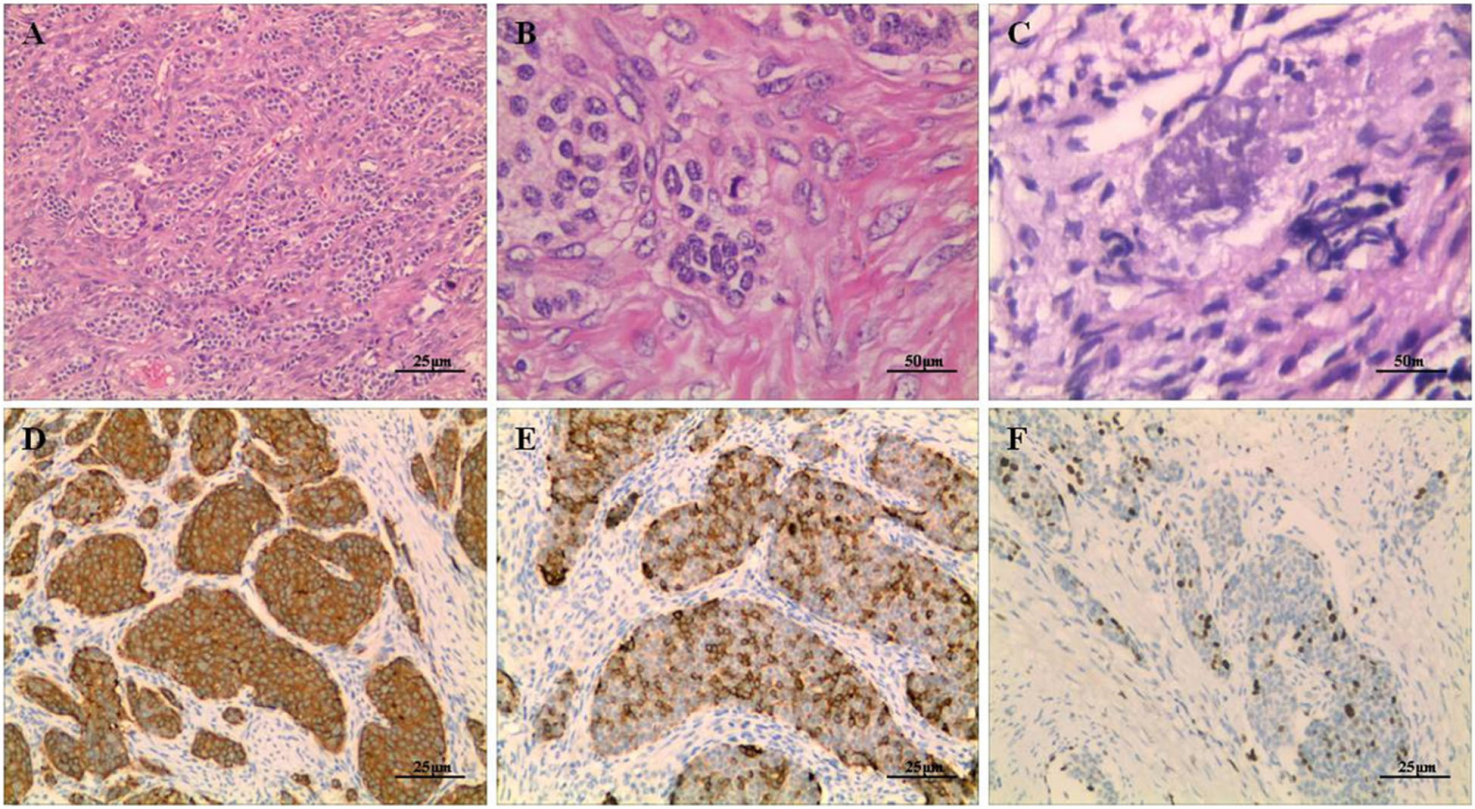
Histological examination of the surgical specimen of the ovary showed that the main part of the tumor had a trabecular pattern, but an insular pattern was also observed in some sections of the tumor, original magnification, × 200 (**a**), more than 2 mitosis per 10 high-power field, original magnification, × 400 (**b**) and foci of necrosis, original magnification, × 400 (**c**). Immunohistochemistry analysis of a tumor sample from the right ovary demonstrating positive detection of synaptophysin, original magnification, × 200 (**d**), CgA, × 200 (**e**), and staining index for Ki-67 was between 10 and 15%, × 200 (**f**)

For diagnostic purposes, the patient underwent a needle biopsy of the second rib. Microscopic examination revealed that the tumor had invaded the bone and consisted of cellular nests of small, round epithelial cells. The tumor had a distinct and identifiable neuroendocrine growth pattern which consisted of various architectural types. Most tumor cells were positive for synaptophysin and the Ki-67 nuclear labeling index was + 1% (Fig.4). The histological diagnosis was metastases from a neuroendocrine tumor to the rib. To confirm the primary tumor, her left oophorectomy records indicating a trabecular carcinoid diagnosis made at an outside institute were retrieved and reviewed. Microscopically, the main part of this previous tumor had a trabecular pattern, however, an insular pattern was also observed in some sections. A few sections showed a higher number of atypical nuclei, a higher mitotic rate, and focal necrosis of tumor cells. Most tumor cells were positive for synaptophysin and Ki-67 was approximately 15% (Fig. 5). The tumor cells were negative for TTF-1, PAX-8, CDX-2, CD99, inhibin-α, and calretinin. The morphological appearance, positive neuroendocrine markers, and intermediate Ki-67 index all suggested a neuroendocrine tumor of the atypical carcinoid subtype. Notably, her original diagnosis was a trabecular carcinoid following her first surgery. Two years after the initial diagnosis, metastasis to the contralateral ovary was observed as an incidental finding during a caesarean section.

Laboratory evaluation revealed a calcium level of 2.17 mmol/L (range: 2.11–2.52 mg/dl). Her presenting parathyroid hormone (iPTH) levels were 33.90 pg/ml (range: 12–88 pg/ml). Serum thyroid hormone levels were normal. Based on parathyroid, thymic, pancreatic, and adrenal gland tumor involvement, the patient was diagnosed with MEN1. She began treatment with everolimus, and octreotide therapy was planned.

## Discussion and conclusions

MEN1 is a rare disorder and as result missed or incorrect diagnoses in patients with sporadically occurring MEN1-related tumors are relatively common. MEN1 diagnosis relies on measurement of specific hormones as well as imaging studies of the affected organs. Primary hyperparathyroidism (HPT) is the most common feature and the original manifestation of MEN1 in most cases. Initial diagnostic workup and follow-up are difficult because of the multiplicity of the affected glands which makes measurement of specific hormones difficult. Currently, the simultaneous presence of at least two of the three neuroendocrine tumors (parathyroid, pancreatic islets, or pituitary) has been considered pathognomonic for MEN1. In addition, patients are prone to develop adrenal adenomas/carcinomas, carcinoids (gastric, lung, thymic), and thyroid adenomas [12–14]. Although surgical treatment to remove endocrine tumors and medical therapy to control hormonal hypersecretion are the cornerstone of MEN1 syndrome treatment, it is very difficult to predict the prognosis of patients with MEN1 because of complications associated with affected organs [15]. Currently, approximately two thirds of patients with MEN1 die of causes directly related to MEN1. Duodenopancreatic NETs are the leading cause of mortality because of their malignant behavior [16].

Due to the complex clinical characteristics of MEN1, it is difficult to define the premonitory and first symptoms. To the best of our knowledge, a MEN1-related ovarian carcinoid is a rare finding that has not been previously studied or reported. The potential for widespread metastasis resulting from MEN1-related ovarian carcinoids remains largely unknown. With a morphologically insular growth pattern, the differential diagnosis in the presented case included metastatic neuroendocrine tumors before considering other ovarian primary tumors. The parathyroid gland, thymus, lung, pancreas, and adrenal gland carcinoid tumors, as well as multiple associated metastatic tumors, were detected during a clinical follow-up. Thus, our patient had multiple neuroendocrine tumors, which exceeded the characteristic presentation of at least three neoplasms, of which one was aggressive and had a guarded prognosis. Based on the imaging and pathological findings, she was diagnosed with MEN1. Unfortunately, our patient was not originally suspected of MEN1 despite her bilateral ovarian involvement during a two-year interval. No abnormal hormonal changes were identified and there was no significant family history. Generally, syndromes such as MEN4 and Familial Isolated Pituitary Adenomas (FIPA) can predispose patients to neuroendocrine tumors and can therefore cause a MEN1-like phenotype; these need to be considered in mutation- negative patients [17].

Results of previous studies suggested that thymic (TH) and pancreaticoduodenal (PD) neuroendocrine tumor (NETs) have poor outcomes and should be diagnosed early [16, 18, 19]. Therefore, some studies recommend a yearly chest CT in all patients with MEN1 who are older than 25 years which would allow for early detection of these tumors. If MEN1 had been suspected or diagnosed at the time of the prior oophorectomies in this young patient, the clinical management, disease course, and patient outcomes would have been dramatically different, and the bone metastases would have potentially been avoided or at least delayed. Physicians should be aware and perform proper assessments of these tumors to prevent delays in diagnosis since MEN1 treatment is reliant on the affected gland(s) and underlying hormonal syndromes.

Carcinoid tumors usually metastasize to the lungs, liver, and lymph nodes [20]. Interestingly, this case had widespread skeletal metastases including the skull, and to our knowledge, widespread skeletal metastasis with MEN1 has not been reported. Skeletal metastases from carcinoid tumors are considered to be exceedingly rare [21], and the most common site of metastasis is the spine [16]. Previous retrospective studies have revealed that the incidence of bone metastases is between 7 and 15% when octreotide scintigraphy is used to evaluate carcinoid tumors [22]. However, a study the examined autopsy results found a higher rate (42%) of skeletal metastases in 36 patients with carcinoid tumors [23].

In most cases, the patients were examined for skeletal metastases since they had clinical symptoms suggestive of metastasis. However, patients with carcinoid tumors and skeletal metastases do not always complain of pain at the metastatic sites [22]. Even if patients do not specifically complain of any metastasis related symptoms, metastasis should nevertheless still be considered. Bone metastases pose a considerable risk for complications including pathological fractures, spinal cord compression, and, rarely, hypercalcemia. These complications result in a reduced patient quality of life. Therefore, when patients present with carcinoid tumors, they should be followed up carefully since skeletal metastasis is an adverse prognostic factor for patients with MEN1. Given the complex manifestations of MEN1, confirmatory genetic testing should be recommended for patients when disease is first suspected. However, in our case that patient did not undergo genetic testing due to the associated costs. Patients with confirmed MEN1 should be advised to undergo periodic biochemical screening in addition to regular medical imaging. In the current case, timely MEN1 diagnosis and regular follow-up may have allowed for earlier detection of the carcinoid tumors and metastases.

We reported a very rare case of atypical ovarian carcinoid that presented as the initial manifestation of early age onset MEN1. It is significant to note that widespread skeletal metastasis of carcinoid tumors can occur in patients with MEN1, even in those without noticeable symptoms. Timely diagnosis of MEN1 is necessary to enable effective treatment and patients should undergo regular follow-up to ensure adequate control of disease. Future studies focused on comparing the clinical manifestation and outcomes of MEN1 patients in addition to evaluating long-term follow-up data are needed to more accurately describe the clinical picture of this disease.

## Data Availability

Data sharing is not applicable to this article since no datasets were generated or analyzed for this study.

## Abbreviations

CT: Computed tomography
HPT: Primary hyperparathyroidism
MEN1: Multiple endocrine neoplasia type 1 syndrome
NETs: Neuroendocrine tumors
PD: Pancreaticoduodenal
PET-CT: Positron emission tomography–computed tomography
PTH: Parathyroid hormone
TH: Thymic
